# Resistance to Antibiotics of Clinical Relevance in the Fecal Microbiota of Mexican Wildlife

**DOI:** 10.1371/journal.pone.0107719

**Published:** 2014-09-18

**Authors:** Jurgi Cristóbal-Azkarate, Jacob C. Dunn, Jennifer M. W. Day, Carlos F. Amábile-Cuevas

**Affiliations:** 1 Division of Biological Anthropology, University of Cambridge, Cambridge, United Kingdom; 2 Center for Conservation Biology, Department of Biology, University of Washington, Seattle, Washington, United States of America; 3 Fundación Lusara, Mexico City, Mexico; University Medical Center Utrecht, Netherlands

## Abstract

There are a growing number of reports of antibiotic resistance (ATBR) in bacteria living in wildlife. This is a cause for concern as ATBR in wildlife represents a potential public health threat. However, little is known about the factors that might determine the presence, abundance and dispersion of ATBR bacteria in wildlife. Here, we used culture and molecular methods to assess ATBR in bacteria in fecal samples from howler monkeys (*Alouatta palliata*), spider monkeys (*Ateles geoffroyi*), tapirs (*Tapirus bairdii*) and felids (jaguars, *Panthera onca;* pumas, *Puma concolor*; jaguarundis, *Puma yagouaroundi*; and ocelots, *Leopardus pardalis*) living freely in two regions of the Mexican state of Veracruz under different degrees of human influence. Overall, our study shows that ATBR is commonplace in bacteria isolated from wildlife in southeast Mexico. Most of the resistances were towards old and naturally occurring antibiotics, but we also observed resistances of potential clinical significance. We found that proximity to humans positively affected the presence of ATBR and that ATBR was higher in terrestrial than arboreal species. We also found evidence suggesting different terrestrial and aerial routes for the transmission of ATBR between humans and wildlife. The prevalence and potential ATBR transfer mechanisms between humans and wildlife observed in this study highlight the need for further studies to identify the factors that might determine ATBR presence, abundance and distribution.

## Introduction

Antibiotic production and, therefore, antibiotic resistance (ATBR) are ancient phenomena [1], [2]. However, the current variety of resistant organisms, their geographic distribution, and the breath of resistance in single organisms in the clinical setting are unprecedented and mounting [3]. The growing number of reports of antibiotic resistant bacteria (usually *Escherichia coli* and enterococci) in wildlife [4]–[10] is also a cause for concern, as they include resistance towards drugs that are commonly used in hospitals (e.g., extended-spectrum beta-lactamase-mediated resistance to third-generation cephalosporins [11]). Moreover, resistance towards synthetic antibiotics (such as fluoroquinolones), which cannot have been selected by ancient, naturally-occurring antibiotics, has also been reported in wildlife [12].

Previous work has shown that resistant microorganisms in wildlife tend to be more abundant closer to human settlements [10], [13]–[17]. Accordingly, their presence in assumedly antibiotic-free environments has been interpreted as the result of human-mediated dispersal of resistant bacteria, resistance genes, antibiotics and/or other selective pressures, such as heavy metals [18]. In this sense, differences in diet and activity among host species may play an important role in determining ATBR in wildlife, as some species come in to more frequent contact with humans, human landscapes, or domestic animals than others [19]–[21]. However, very few studies have traced resistance genes found in antibiotic-free environments directly to human sources [22] and we know very little about what might lead to the development of ATBR in wildlife in areas outside of direct human contact. Such data are needed to understand the complexity of the ATBR phenomenon in wildlife, and to extend our knowledge beyond the simplistic notions that antibiotic abuse is the only driver of bacterial resistance and that diminishing antibiotic usage will, therefore, reduce it. Furthermore, given that 60% of emerging infectious diseases are zoonoses, of which 70% originated in wildlife, ATBR in wildlife represents a potential public health threat [23]. Therefore, there is an urgent need to assess the resistance towards antimicrobial agents in wildlife and the factors that might determine its presence, abundance and dispersion.

Here, we used culture and molecular methods to assess ATBR in bacteria in the fecal microbiota of howler monkeys, spider monkeys, tapirs and felids (jaguars, pumas, jaguarundis, and ocelots) living freely in two regions of the Mexican state of Veracruz under different degrees of human influence. Our objectives were twofold: 1) to characterize the ATBR present in these species and 2) to analyze the effects of environmental characteristics and animal behavior on the distribution of ATBR in wildlife. We predicted that higher levels of habitat disturbance and greater proximity to humans would both be related to higher levels of ATBR, and that terrestrial animals, particularly felids, would harbor a higher and more diverse number of resistance phenotypes than arboreal animals, due to the greater level of contact they have with humans and domestic animals.

## Materials and Methods

### Ethics statement

This research was undertaken in accordance with the ethical and legal requirements of the Ministry of Environment and Natural Resources of Mexico (SEMARNAT), and was authorized by permit number SGPA/DGVS/07120/09. The University of Washington Institutional Animal Care and Use Committee approved the use of domestic dogs as part of our research team (protocol 2850-08).

### Study sites

**Figure 1:**
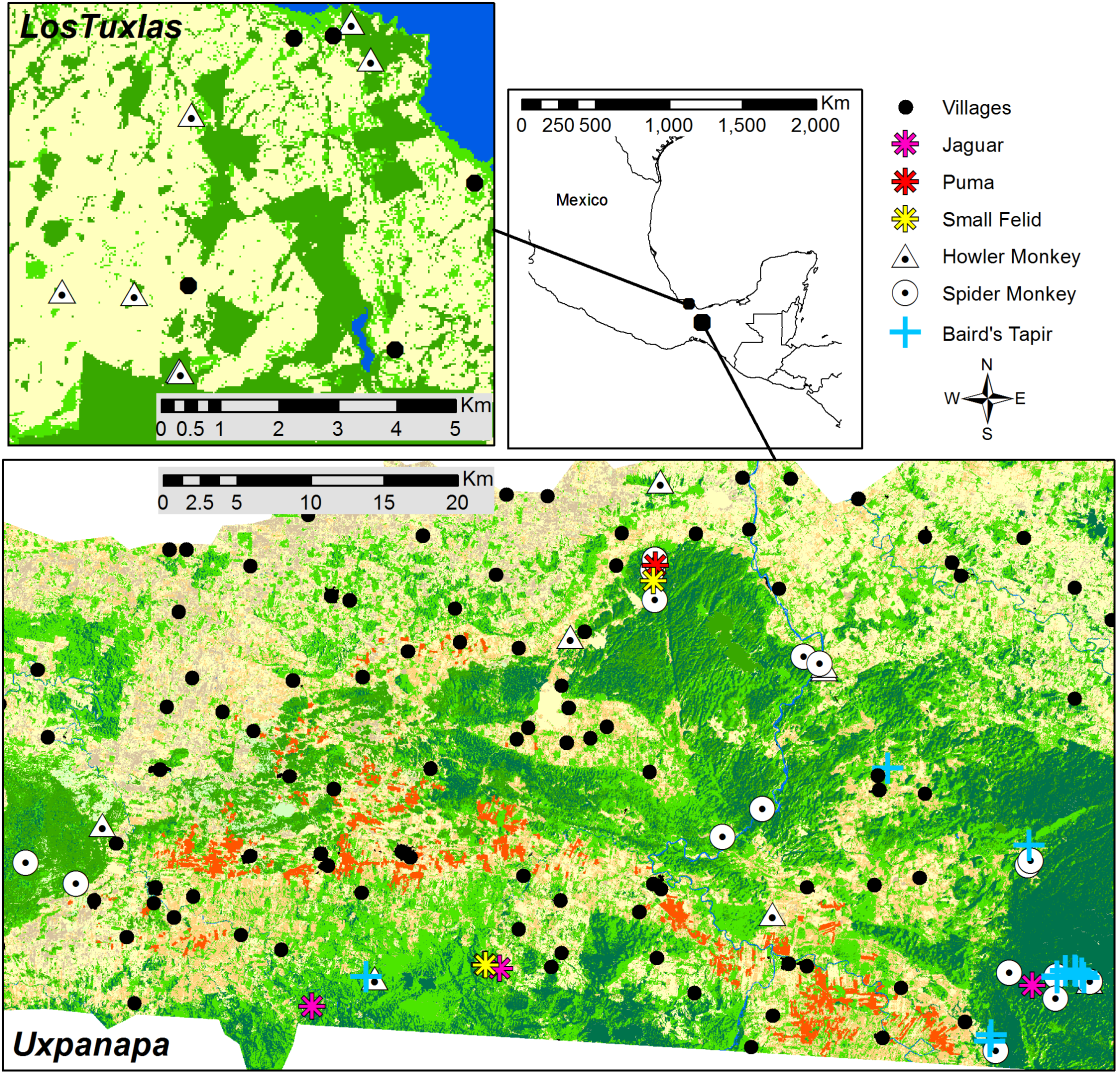
Map showing the locations of fecal samples collected from primates, felids and tapirs in two study sites (Los Tuxtlas and Uxpanapa) in south-east Mexico. Villages are indicated with black dots. Dark green represents mature forest, light green secondary forest, yellow pasture, light brown citric plantations, and red rubber plantations. Los Tuxtlas supervised classification map based on freely available Landsat 2011 images (source: usgs.gov). Uxpanapa supervised classification map based on SPOT5 scenes obtained from ERMEX/SEMAR (2010; source: ERMEXS, Estación de Recepción México de la Constelación SPOT/Secretaría de Marina Armada de México. 2010. SPOT5 images).

We collected fecal samples from March to June 2010 during surveys of free-ranging populations of howler monkeys (*Alouatta palliata*) in Los Tuxtlas, and of howler monkeys (*Alouatta palliata*), spider monkeys (*Ateles geoffroyi*), felids [jaguars (*Pantera onca*), pumas (*Puma concolor*), jaguarundis (*Puma yagouarundi*), and ocelots (*Leopardus pardalis*)], and tapirs (*Tapirus bardii*) in Uxpanapa, which are both regions in the state of Veracruz, Mexico (Figure 1).

Los Tuxtlas is located near the south of the state of Veracruz. The region has a long history of human occupation, dating back over 1,000 years to the earliest Mesoamerican civilization, the Olmecs [24]. The original forest of this region has been extensively transformed into pasture and agricultural landscapes. In the northern part of the region, where we conducted our sampling (18°28′−18°39′N, 93°02′−95°18′W), only 13% of the 75,000 ha of original rainforest remains and the landscape is composed of an archipelago of different sized forest patches that vary in degree of isolation and habitat quality [25]. The human population density in the region is 108.8 inhabitants/ha [26].

Uxpanapa is located approximately 150 km south east of Los Tuxtlas (17°04′−17°31′N, 93°46′−94°49′E); it is the northern limit of the Zoque Forest, which, at over 1,000,000 ha, is the largest remaining tract of tropical rainforest in Mexico. Compared to Los Tuxtlas, the region has a recent history of human occupation, starting in the late 1960’s [27]. Population density in this region is much lower (11.6 inhabitants/ha) [26] and, although deforestation in the region has been extensive in the past 40 years, large tracks of pristine tropical rainforest are still found there, inhabited by a diverse range of animal species.

### Fecal sample collection and transport

Howler and spider monkeys are almost exclusively arboreal and occupy the mid and high strata of the forest. We observed these primates while walking along forest paths, often being alerted to their presence by their vocalizations and movement. Once a group was located, the sampling team waited for them to defecate, thus confirming the origin of the sample. Felids and tapirs are far more elusive and we did not directly observe them during our surveys. Therefore, we located their fecal samples with the assistance of a trained scat detection dog [28]. Scat detection dogs are able to find samples from multiple species simultaneously across large, remote areas and have a lower sampling bias than traditional wildlife detection methods [28]–[30]. When the dog detects the scat, it signals its whereabouts to the handler by sitting a short distance away from the sample. Therefore, the samples are not contaminated by the presence of the dog.

We collected fresh fecal samples in transport swabs containing Stuart transport agar (Copan Diagnostics) only if free from environmental contamination (e.g., dust, mud). We kept samples on ice packs until refrigeration, which was no later than 4 days after sample collection. Additionally, for the terrestrial mammals (i.e., felids and tapirs), we collected duplicate samples for genetic analysis by swabbing the surface of the scat with sterile foam swabs soaked in phosphate buffered saline (PBS) solution. Genetic samples were then stored either in 90% ethanol (and later dried with desiccant) or in lysis buffer, and kept in a refrigerator, freezer, or on dry-ice in the field, as available.

### Genetic typing of tapir and felid samples

We used mitochondrial DNA (mtDNA) markers to confirm the species origin of the felid and tapir samples, since felid scats cannot be distinguished from each other visually, and tapir feces can be confused with horse/donkey feces. Swabbing the surface of the scat samples minimizes downstream PCR inhibitors and maximizes epithelial cell DNA [31]. DNA was extracted from the swab samples using the tissue extraction protocol of the Qiagen Tissue Kit (Qiagen Inc.). We used a 175-bp sequence of the ATP6 ribosomal subunit gene [32], [33], which lays outside of the felid *numt* region that renders many mtDNA markers unreliable for felids. We then established the preliminary species assignment using NCBI’s BLAST search and we further confirmed this by aligning the sequences with in-house control sequences using the MEGA5 software [34]. For further corroboration of felid species identification, we used a second molecular marker, a species-specific fragment polymorphism of a different mtDNA region amplified using the HSF21 and LTPROB13 primers [35].

### Detection and isolation of antibiotic resistant strains

We re-suspended swabs in 1 mL PBS by vigorous mixing, then plated 50 µL of the suspension on Mueller-Hinton plates containing antibiotics (ampicillin, sulfamethoxazol, nalidixic acid, gentamicin, tetracycline and chloramphenicol). We also plated 50 µL of the suspension on eosin-methylene-blue (EMB) agar to check for viability of enteric bacteria. We incubated plates aerobically at 35°C for 24 h. We isolated the colonies grown on antibiotic plates on antibiotic-free medium for further analyses, including identification based on gram staining and standard biochemical techniques.

### Antibiotic susceptibility testing

We tested susceptibility towards 8 antibiotics: ampicillin (AM), amoxicillin-clavulanate (AMC), cefotaxime (CTX), gentamicin (G), tetracycline (TE), chloramphenicol (CML), ciprofloxacin (CIP) and sulfadiazine (SUL) using the disk diffusion method (BBL disks on Mueller-Hinton agar). We interpreted the resulting inhibitory halos according to Clinical and Laboratory Standards Institute guidelines. Following Över et al. [36], we applied further antibiotic susceptibility testing to all G-resistant isolates, which included assaying a set of 12 aminoglycoside compounds designed to assess the underlying mechanism of resistance. Briefly, we used disks containing 12 different aminoglycosides (6 of them not used clinically) for typical disk-diffusion susceptibility testing. Each known aminoglycoside-modifying enzyme, and most common combinations, yield a distinct inhibitory halo profile, while a uniform reduction of the activity of all 12 is interpreted as a result of decreased permeability. Although varying levels of enzyme expression can produce atypical profiles, the method can reliably distinguish between enzyme-mediated and permeability-mediated aminoglycoside resistance, which was the main goal here.

### Integron PCR assays

Class I integrons are bacterial genetic elements that play a role in the acquisition and dissemination of antibiotic resistance genes. Little is known about the distribution or abundance of integrons outside of the clinical context [13]. However, evidence suggests that the prevalence of class 1 integrons is directly related to exposure to human environments [37]. Therefore, we attempted to amplify the *intI1* integrase gene of class-1 integrons for all *E. coli* isolates using the PCR primers (intI1.F: 5′-GGGTCAAGGATCTGGATTTCG-3′; and intI1.R: 5′-ACATGGGTGTAAATCATCGTC-3′) and conditions reported in [38].

### Environmental characteristics

We recorded the location of each sample using a GPS unit. In Uxpanapa, we later calculated the shortest distance to the nearest anthropogenic habitat (pasture, plantation, orchard, etc.), shortest distance to the nearest human settlement, and the number of human settlements within a 2.5 km, 5 km and 10 km radius, using a classified 2008 Landsat satellite image (1∶20,000 scale) of the study area, the ArcView GIS software (version 3.1), and the Patch Analyst 2.2 extension for ArcView [39]. We did not perform these analyses for the samples collected in Los Tuxtlas given that they all belonged to groups of howler monkeys living in forest fragments in close proximity to human settlements (within 1 km distance).

### Statistical analysis

We calculated four ATBR parameters: 1) the proportion of samples resistant to at least one antibiotic (rS); 2) the number of isolates per sample that where resistant to at least one of the drugs tested (rO); 3) the total number of antibiotic resistance phenotypes detected per sample (rP); and 4) the average number of antibiotics each isolate per sample was resistant to (rA). For example, if a sample had two different isolates, an *Escherichia coli* and a *Pseudomononas* sp., and the first isolate was resistant to AM and SUL, and the second to CIP, CML and G, the resistance parameters for this sample would have been: rO = 2, rP = 5 and rA = 2.5. We excluded resistance phenotypes deemed “intrinsic” (i.e., not being selected by antibiotics: AM, AMC and CTX resistance in *Pseudomonas*; AM and AMC resistance in *Acinetobacter*; CTX resistance in enterococci), from all calculations. We included in our analysis those traits that are usually thought of as “intrinsic resistance”, such as chromosomally-encoded AmpC beta-lactamases in *Enterobacter* and *Klebsiella*, and aminoglycoside-resistance due to decreased accumulation in *Pseudomonas aeruginosa*, if they were not present in all our isolates of a given taxon, as this would suggest that they were selected for by antibiotics or related agents. Also, we calculated the prevalence of different ATBR for each host species, that is, the number of samples resistant to a given antibiotic. Finally, since we did not process swabs to reveal the total composition of the fecal microbiota, but only to isolate resistant organisms, we were unable to assess the resistance rate per bacterial species. However, since *E. coli* was present in all samples, as inferred from the characteristic metallic green hue on EMB plates, we were able to determine the prevalence of resistance in this species (Table 1).

**Table 1:** Antibiotic resistance prevalence (N [%]) in *E. coli* isolates.

Host	Location	AM	AMC	CML	SUL	CIP	TE	N
Howler	Los Tuxtlas	6 [11]	1 [2]	0 [0]	4 [7]	0 [0]	2 [3]	60
Howler	Uxpanapa	6 [24]	2 [8]	0 [0]	3 [12]	1 [4]	3 [12]	25
Spider	Uxpanapa	8 [25]	2 [6]	1 [3]	2 [6]	1 [3]	2 [6]	32
Tapirs	Uxpanapa	5 [33]	0 [0]	0 [0]	4 [27]	0 [0]	6 [40]	15
Felids	Uxpanapa	0 [0]	0 [0]	0 [0]	1 [14]	0 [0]	1 [14]	7

N represents the total number of samples analyzed.

We used a Z test for proportions (independent groups) to compare rS among howler monkey samples collected in Los Tuxtlas and Uxpanapa, among samples from different species, between terrestrial and arboreal species in Uxpanapa, and between samples collected ≤2.5 km away from humans settlements and >2.5 km away. Due to the non-parametric nature of rO, rP and rA, for samples in our study, we used a Mann-Whitney U test to compare these parameters between samples collected ≤2.5 km from a human settlement and those collected further away in Uxpanapa, and between howler monkey samples collected in Los Tuxtlas and Uxpanapa. For the samples collected in Uxpanapa, we used a Kruskal-Wallis H test to analyze the existence of overall differences in rO, rP and rA among the different study species, and a Mann-Whitney U test to conduct pairwise comparison of these parameters between the different species, as well as to compare terrestrial and arboreal species. Finally, we analyzed the relationship between rO, rP and rA parameters and the distance to the nearest human settlement and pasture in Uxpanapa using linear regression analyses. All analyses were carried out in SPSS Version 20.0, considering *p*<0.05 as significant.

## Results

We collected a total of 138 fecal samples: 85 samples from howler monkeys (25 samples from 8 groups in Uxpanapa and 60 samples from 7 groups in Los Tuxtlas), 32 samples from 18 groups of spider monkeys, 14 samples from tapirs, and 7 samples from felids. The geographical distribution of these samples is shown in Figure 1.

### Resistance prevalence for howler monkeys

#### Los Tuxtlas

Resistance prevalence for antibiotics was: AM = 51%, AMC = 24%, TE = 43%, C = 36%, SUL = 19%, G = 13%, CTX = 5%. We did not detect any resistance to CIP. We considered three *Pseudomonas* spp. isolates to express an extended-spectrum beta-lactamase (ESBL), judging from the inhibitory halos around CTX and AMC [40]. One *E. coli* isolate, resistant only to SUL, carried an *intI1* gene. We considered three out of 12 (25%) G-resistance phenotypes to be mediated by aminoglycoside-modifying enzymes. Forty-five isolates (48%) were resistant to a single antibiotic, 25 (27%) to two, 15 (16%) to three, 5 (5%) to four and 4 (4%) to five. Isolated resistant bacteria were: 24 *Pseudomonas* spp. (26%), 12 *E. coli* (13%), 12 *Enterobacter* spp. (13%), 10 *Acinetobacter* spp. (11%), 10 *Citrobacter* (11%), 9 *Klebsiella* spp. (10%), 6 other Enterobacteriaceae (6%), 4 other non-fermentative (4%), and 7 gram-positives (7%). Eleven percent of *E. coli* isolates were resistant to AM, 2% to AMC, 0% to CML, 7% to SUL, 0% to CIP and 3% to TE (Table 1).

#### Uxpanapa

Resistance prevalence for antibiotics was: AM = 76%, AMC = 47%, TE = 47%, C = 6%, SUL = 12%, G = 15%, CTX = 3%, CIP = 6%. We considered 5 isolates, 4 *Acinetobacter* spp. and one *Citrobacter* spp. to express an ESBL. We found no *intI1* gene among *E. coli* isolates. Ten isolates (29%) were resistant to a single antibiotic, 12 (35%) to two, 10 (29%) to three, and 2 (6%) to four. Isolated resistant bacteria were: 10 *Enterobacter* spp. (29%), 7 *E. coli* (21%), 7 *Klebsiella* spp. (21%), 5 *Acinetobacter* spp. (15%), and 4 *Citrobacter* spp. (12%). Resistance prevalence of *E. coli* isolates to different antibiotics was: 24% AM, 8% AMC, 0% CML, 12% SUL, 4% CIP and 12% TE (Table 1).

### Resistance prevalence for spider monkeys

Resistance prevalence for antibiotics was: AM = 85%; AMC = 50%; TE = 35%; C = 8%; SUL = 8%; G = 5%; CTX = 3%, and CIP = 2%. We considered two isolates, an *Enterobacter* spp. resistant to AM, AMC and CTX and an *Acinetobacter* spp. resistant to G, to express an ESBL. One *E. coli* isolate, resistant to AM, SUL, and TE, carried an *intI1* gene. We considered one G-resistance phenotype to be mediated by aminoglycoside-modifying enzymes. Twenty-two isolates (37%) were resistant to a single antibiotic, 20 (33%) to two, 17 (28%) to three, and 1 (2%) to five. Isolated resistant bacteria were: 28 *Enterobacter* spp. (47%); 8 *E. coli* (13%), 9 *Klebsiella* spp. (15%); 3 *Citrobacter* spp. (5%); 4 other fermentative bacteria (7%); 6 *Pseudomonas* spp. (10%); 2 *Acinetobacter* spp. (3%); 1 gram-positive (2%). Resistance prevalence of *E. coli* isolates to different antibiotics was: 25% AM, 6% AMC, 3% CML, 6% SUL, 3% CIP and 6% TE (Table 1).

### Resistance prevalence for tapirs

Genetic typing confirmed that 14 fecal samples belonged to tapirs. Resistance prevalence for antibiotics was: AM = 59%; TE = 46%; C = 23%; SUL = 15%; G = 8%; AMC = 15%; CTX = 3%; with no CIP-resistant organism or ESBL. We considered three G-resistance phenotypes to be mediated by aminoglycoside-modifying enzymes. One *E. coli* isolate, resistant to SUL and TE, carried an *intI1* gene. Twenty isolates (51%) were resistant to a single antibiotic, 14 (36%) to two, 3 (8%) to three, one (3%) to four and one (3%) to five. Isolated resistant bacteria were: 3 *Enterobacter* spp. (8%); 9 *E. coli* (23%), 2 *Klebsiella* spp. (5%); 4 *Citrobacter* spp. (10%); 3 other fermentative bacteria (8%); 8 *Pseudomonas* spp. (21%); 2 *Acinetobacter* spp. (5%); one other non-fermentative bacteria (3%); 7 gram-positives (18%). Resistance prevalence of *E. coli* isolates to different antibiotics was: 33% AM, 0% AMC, 0% CML, 27% SUL, 0% CIP and 40% TE (Table 1).

### Resistance prevalence for felids

Genetic typing confirmed that 7 samples were felids (3 jaguars, 1 puma, 1 ocelot and 2 jaguarundis). Resistance prevalence for antibiotics was: AM = 62%; TE = 29%; C = 19%; AMC = 19%; G = 24%; SUL = 14%; CTX = 10%; with no CIP-resistant organisms or ESBL. We also found no *intI1* gene among *E. coli* isolates. We observed one G-resistance phenotype, which we considered to be mediated by aminoglycoside-modifying enzymes. Nine isolates (43%) were resistant to a single antibiotic, 9 (43%) to two, and 3 (14%) to three. Isolated resistant bacteria were: 4 *Enterobacter* spp. (19%); 1 *Escherichia coli* (5%); 2 *Klebsiella* spp. (10%); 2 *Citrobacter* spp. (10%); 5 other fermentative bacteria (24%); one *Pseudomonas* spp. (5%); 1 *Acinetobacter* spp. (5%); 2 other non-fermentative bacteria (7%), 3 gram-positives (14%). Resistance prevalence of *E. coli* isolates to different antibiotics was: 0% AM, 0% AMC, 0% CML, 14% SUL, 0% CIP and 14% TE (Table 1).

### Human influence on ATBR parameters

On average, the ATBR parameters rS, rO and rP where higher in the samples from the howler monkeys living in the more anthropogenically disturbed Los Tuxtlas region (83%; 1.6±1.1; 3.0±2.7, respectively) than in the more conserved Uxpanapa region (76%; 1.4±1.1; 2.9±2.8, respectively), while the opposite was true for rA (Table 2). However, these differences where not statistically significant.

**Table 2:** Resistance parameters in different host categories, including howler monkeys from two locations and four mammal taxa (two arboreal and two terrestrial) in the same location (Uxpanapa).

Host	Location	(rS)	(rO)	(rP)	(rA)	N
Howler monkey	Los Tuxtlas	83%	1.6±1.1	3.0±2.7	1.9±1.1	60
Howler monkey	Uxpanapa	76%	1.4±1.1	2.9±2.8	2.1±0.9	25
Spider monkey	Uxpanapa	94%	1.9±1.0	3.7±2.1	2.0±0.9	32
Tapir	Uxpanapa	100%	2.6±1.0	4.3±2.4	1.7±0.9	15
Felids	Uxpanapa	100%	3.0±1.7	4.3±2.5	1.7±0.7	7
Arboreal	Uxpanapa	85%	1.7±1.1	3.4±2.5	2.0±1.0	55
Terrestrial	Uxpanapa	100%	2.7±1.2	4.3±2.4	1.7±0.8	22

N represents the total number of samples analyzed.

Considering all the samples from all host species in Uxpanapa, regression analysis revealed no significant relationship between any of the ATBR parameters and either the distance to nearest anthropogenic habitat or the distance to the nearest human settlement. However, samples collected at a distance ≤2.5 km from a human settlement in this region had significantly higher rO (1.11±0.96 vs. 2.13±1.17 isolates; U = 827.0, *p* = 0.002, df = 1) and rP (2.05±1.76 vs. 4.0±2.41; U = 826.0, *p* = 0.003, df = 1) than those collected further away. However, there was no significant difference in rA (1.85±0.87 vs. 1.92±0.91) or rS (87% vs. 95%).

### Differences in ATBR parameters in Uxpanapa

Among the study species in Uxpanapa, rS was highest in felids and tapirs (100%), followed by spider monkeys (94%) and howler monkeys (83%). However, these values were only significantly different between howlers and tapirs (*p* = 0.046), and marginally significant between howler and spider monkeys (*p* = 0.063) (Table 2). When we compared rS between terrestrial and arboreal species, this was higher in terrestrial (85 vs. 100%) (Table 1), but the difference was only marginally significant (*p* = 0.058).

We found significant differences in rO among species (H = 13.29, *p* = 0.004, df = 3), this being highest in felids (3.0±1.7), followed by tapirs (2.6±1.0), spider monkeys (1.9±1.0) and howler monkeys (1.4±1.1) (Table 2). Pairwise comparison showed that these differences were significant between howler monkeys and all other taxa (spider monkeys: U = 521.5, *p* = 0.041, df = 1; felids: U = 136, *p* = 0.026, df = 1; tapirs: U = 275, *p* = 0.002, df = 1), and spider monkeys and tapirs (U = 302.5, *p* = 0.049, df = 1). Finally, rO was significantly higher in terrestrial (felids and tapirs) than in arboreal species (howler and spider monkeys) (U = 866.5, *p* = 0.0002, df = 1).

We found rP levels to also be highest in felids (4.3±2.5) and tapirs (3.7±2.1), followed by primates (howler monkeys = 3.0±2.7 and spider monkeys = 2.9±2.8) (Table 2). Overall, these differences where not statistically significant, although the difference in rP between howler monkeys and tapirs was close to significance (U = 302.5, *p* = 0.058, df = 1). Similar to rS and rO, rP was significantly higher in terrestrial species than arboreal species (U = 2264.0, *p* = 0.022, df = 1).

Following the opposite trend, rA was highest in howler monkeys (2.1±0.9), followed by spider monkeys (2.0±0.9), tapirs (1.7±0.9) and felids (1.7±0.7) (Table 2). However, none of these differences reached statistical significance. Finally rA was higher in arboreal (2.0±1.0) than in terrestrial species (1.7±0.8), but this difference was not significant.

## Discussion

In this study, we found antibiotic resistance to be commonplace in fecal bacteria from terrestrial and arboreal wildlife in Mexico. This is consistent with other studies on ATBR genes and phenotypes in bacteria collected from wildlife and wild settings [4], [41]. Overall, the great majority of the resistance phenotypes detected where to old, naturally-occurring antibiotics. However, we also found resistance to synthetic (CIP) and semi-synthetic (CTX, ESBL-mediated) antibiotics, which are not expected to be present in environments without significant human influence, and class-1 integrons, that have also been directly linked to anthropogenic influence [37].

In accordance with our first prediction, we found that proximity to human settlements was associated with higher levels of several ATBR parameters. Overall, ATBR was higher in howler monkeys from Los Tuxtlas than those from Uxpanapa, and traits likely to be mobile, such as enzyme-mediated G-resistance and plasmid-mediated beta-lactamases, as well as class-1 integrons, were only found in howler monkeys from the more disturbed Los Tuxtlas region. However, resistance prevalence in *E. coli* was consistently higher in isolates from howler monkeys from Uxpanapa than those from Los Tuxtlas (although most AM-resistance in Los Tuxtlas was likely plasmid-mediated: 53% of AM-resistant were AMC-susceptible in Los Tuxtlas, vs. 38% in Uxpanapa). Furthermore, resistance to CIP, a synthetic antibiotic, was only found in monkeys from the better-conserved region (Uxpanapa); and ESBLs were found in howler and spider monkeys from both Los Tuxtlas and Uxpanapa. Although the differences in ATBR parameters between sites did not reach statistical significance, they show similar tendencies across most tests and we think this is probably an effect of small sample size.

In line with our second prediction, this study suggests that the terrestrial species were more exposed to antibiotics from human origin, and/or bacteria from humans and livestock than the arboreal species. Both felids and tapirs frequently leave the forest and travel across pastures, and pumas and jaguars also occasionally prey on cattle. On the other hand, humans and livestock also defecate into the forest, which expose terrestrial wildlife to their bacteria. All this would facilitate the transmission of ATBR between humans/livestock and wildlife and constitute a terrestrial route for the spread of ATBR. Nevertheless, we cannot exclude the possibility that higher ATBR abundance in terrestrial species may also be caused by naturally occurring selective pressures for ATBR being confined to the soil. It is also important to note that we collected far fewer samples from terrestrial animals than arboreal animals, which affects our ability to adequately assess the differences between arboreal and terrestrial taxa. Therefore, further sampling is necessary to confirm this hypothesis.

There are several ways in which the arboreal species may have come into contact with ATBR bacteria, ATBR genes, and/or antibiotics. Firstly, both howler monkeys and spider monkeys do occasionally descend to the ground, particularly in highly fragmented landscapes [42], [43]. Secondly, species that use both the arboreal and the terrestrial strata, such as coatis, might be functioning as vectors. However, the fact that only isolates from primates presented ESBLs and CIP-resistance, suggest the existence of a second aerial route of transmission of ATBR in primates. As these traits are typical of clinical settings (although low-level CIP-resistance might be selected by non-antibiotic agents in the environment [44]), it is very unlikely that they came from nearby settlements. However, ESBLs have been found in enterobacteria from free-living gulls from Alaska [45], among other wildlife. ATBR bacteria and genes have previously been isolated in birds and bats, which could be acting as vectors between humans and wildlife. Birds, especially, seem to be dispersing resistant bacteria generated by the use of antibiotics in food production animals [46]. Both Los Tuxtlas and Uxpanapa are areas of intense bird migratory activity [47], which could be a contributing factor to the ATBR detected in arboreal mammal species.

It is not clear why howler monkeys generally had lower levels of ATBR than spider monkeys. However, both home range area and group size are greater in spider monkeys [48]. This could expose spider monkeys to larger amounts of antibiotics and/or bacteria from human origin, or to more individuals carrying resistant bacteria. On the other hand, spider monkeys may be more prone to descend to the ground, increasing their exposure to ATBR determinants. However, there is little data available regarding the frequencies of these behaviors.

Finally, it is important to emphasize that factors that affect the composition of the microbiota could also modify the prevalence of resistance reported here. For instance, should one kind of animals be prone to carry more *Enterobacter* spp., the prevalence of AM- and AMC-resistance should also rise, as these species commonly carry a chromosomal beta-lactamase. That could be the case for spider monkeys, where nearly half of the isolates were *Enterobacter* spp., and, accordingly, the prevalence of AM and AMC resistance rose significantly. On the other hand, as all four mammals sampled here have different diets, it would be expected that their microbiota is different. Whether the selection of antibiotic-resistance traits affect the composition, or other factors that affect the composition influence the prevalence of resistance, cannot be inferred from these data.

Overall, this study shows that resistance to old, naturally-occurring antibiotics is common in the fecal microbiota of wild mammals. The counterintuitive nature of the data on *E. coli* resistance, that also goes against other resistance indicators used in this study, suggests that *E. coli* might not be a reliable indicator of the human impact on resistance in wildlife bacteria and demonstrates that examining non-*E.coli* species when conducting phenotypical screenings, is essential to get a better picture of ATBR in wildlife [19]. Selective pressures and/or resistant bacteria seem to be more common at ground level; and indicators of ATBR prevalence and mobility seem to increase the closer animals are to human settlements. However, other indicators, such as resistance to synthetic antibiotics, do not seem to follow this trend, as they were only found in isolates from arboreal animals far from human influence. The identification of non-antibiotic pressures that select or maintain ATBR is one of the major gaps in our understanding of the emergence and evolution of these traits. It is also important to realize that human, animal and environmental health are not isolated realms, but a single continuum where factors apparently affecting only one of them, end up having wide repercussions (e.g., www.onehealthinitiative.com). The need for information on the ecological drivers of ATBR in wildlife, its transmission dynamics, and the range of conditions under which gene/bacteria exchange occur is urgent, especially as major pharmaceutical companies have largely abandoned the antibiotic discovery field [49].

## References

[1] D’Costa VM, King CE, Kalan L, Morar M, Sung WWL, et al (2011) Antibiotic resistance is ancient. Nature 477: 457–461 10.1038/nature10388 21881561

[2] Davies J, Davies D (2010) Origins and evolution of antibiotic resistance. Microbiol Mol Biol Rev 74: 417–433 10.1128/MMBR.00016-10 20805405 PMC2937522

[3] Levy SB (2002) The antibiotic paradox: how misuse of antibiotics destroys their curative powers. Cambridge, MA: Perseus Publishing.

[4] Allen HK, Donato J, Wang HH, Cloud-Hansen KA, Davies J, et al (2010) Call of the wild: antibiotic resistance genes in natural environments. Nat Rev Microbiol 8: 251–259 10.1038/nrmicro2312 20190823

[5] Rolland RM, Hausfater G, Marshall B, Levy SB (1985) Antibiotic-resistant bacteria in wild primates: increased prevalence in baboons feeding on human refuse. Appl Environ Microbiol 49: 791–794. 4004213 10.1128/aem.49.4.791-794.1985 PMC238447

[6] Gilliver MA, Bennett M, Begon M, Hazel SM, Hart CA (1999) Antibiotic resistance found in wild rodents. Nature 401: 233–233. 10499578 10.1038/45724

[7] Souza V, Rocha M, Valera A, Eguiarte LE (1999) Genetic structure of natural populations of *Escherichia coli* in wild hosts on different continents. Appl Environ Microbiol 65: 3373–3385. 10427022 10.1128/aem.65.8.3373-3385.1999 PMC91507

[8] Goldberg TL, Gillespie TR, Rwego IB, Wheeler E, Estoff EL, et al (2007) Patterns of gastrointestinal bacterial exchange between chimpanzees and humans involved in research and tourism in western Uganda. Biol Conserv 135: 511–517 10.1016/j.biocon.2006.10.048

[9] Schaefer AM, Goldstein JD, Reif JS, Fair PA, Bossart GD (2009) Antibiotic-resistant organisms cultured from Atlantic bottlenose dolphins (*Tursiops truncatus*) inhabiting estuarine waters of Charleston, SC and Indian River Lagoon, FL. Ecohealth 6: 33–41 10.1007/s10393-009-0221-5 19415386

[10] Thaller MC, Migliore L, Marquez C, Tapia W, Cedeño V, et al (2010) Tracking acquired antibiotic resistance in commensal bacteria of Galápagos land iguanas: no man, no resistance. PLoS One 5: e8989 10.1371/journal.pone.0008989 20126545 PMC2813872

[11] Guenther S, Ewer C, Wieler L (2011) Extended-spectrum beta-lactamases producing *E. coli* in wildlife, yet another form of environmental pollution? Front Microbiol 2: 246. 22203818 10.3389/fmicb.2011.00246 PMC3244693

[12] Literak I, Dolejska M, Janoszowska D, Hrusakova J, Meissner W, et al (2010) Antibiotic-resistant *Escherichia coli* bacteria, including strains with genes encoding the extended-spectrum beta-lactamase and QnrS, in waterbirds on the Baltic sea coast of Poland. Appl Environ Microbiol 76: 8126–8134. 20952638 10.1128/AEM.01446-10 PMC3008254

[13] Hardwick SA, Stokes HW, Findlay S, Taylor M, Gillings MR (2008) Quantification of class 1 integron abundance in natural environments using real-time quantitative PCR. FEMS Microbiol Lett 278: 207–212 10.1111/j.1574-6968.2007.00992.x 18042230

[14] Skurnik D, Ruimy R, Andremont A, Amorin C, Rouquet P, et al (2006) Effect of human vicinity on antimicrobial resistance and integrons in animal faecal *Escherichia coli* . J Antimicrob Chemother 57: 1215–1219 10.1093/jac/dkl122 16581916

[15] Rwego IB, Isabirye-Basuta G, Gillespie TR, Goldberg TL (2008) Gastrointestinal bacterial transmission among humans, mountain gorillas, and livestock in Bwindi Impenetrable National Park, Uganda. Conserv Biol 22: 1600–1607 10.1111/j.1523-1739.2008.01018.x 18717695

[16] Cole D, Drum DJ V, Stallknecht DE, White DG, Lee MD, et al (2005) Free-living Canada geese and antimicrobial resistance. Emerg Infect Dis 11: 935–938. 15963291 10.3201/eid1106.040717 PMC3367595

[17] Walson JL, Marshall B, Pokhrel BM, Kafle KK, Levy SB (2001) Carriage of antibiotic-resistant fecal bacteria in Nepal reflects proximity to Kathmandu. J Infect Dis 184: 1163–1169 10.1086/323647 11598839

[18] Seiler C, Berendonk TU (2012) Heavy metal driven co-selection of antibiotic resistance in soil and water bodies impacted by agriculture and aquaculture. Front Microbiol 3: 399 10.3389/fmicb.2012.00399 23248620 PMC3522115

[19] Rose JM, Gast RJ, Bogomolni A, Ellis JC, Lentell BJ, et al (2009) Occurrence and patterns of antibiotic resistance in vertebrates off the Northeastern United States coast. FEMS Microbiol Ecol 67: 421–431 10.1111/j.1574-6941.2009.00648.x 19187217 PMC5444207

[20] Costa D, Poeta P, Sáenz Y, Vinué L, Rojo-Bezares B, et al (2006) Detection of *Escherichia coli* harbouring extended-spectrum beta-lactamases of the CTX-M, TEM and SHV classes in faecal samples of wild animals in Portugal. J Antimicrob Chemother 58: 1311–1312 10.1093/jac/dkl415 17023496

[21] Sjölund M, Bonnedahl J, Hernandez J, Bengtsson S, Cederbrant G, et al (2008) Dissemination of multidrug-resistant bacteria into the Arctic. Emerg Infect Dis 14: 70–72 10.3201/eid1401.070704 18258081 PMC2600168

[22] Santamaría J, López L, Soto CY (2011) Detection and diversity evaluation of tetracycline resistance genes in grassland-based production systems in Colombia, South america. Front Microbiol 2: 252 10.3389/fmicb.2011.00252 22174707 PMC3237277

[23] Jones KE, Patel NG, Levy MA, Storeygard A, Balk D, et al (2008) Global trends in emerging infectious diseases. Nature 451: 990–993 10.1038/nature06536 18288193 PMC5960580

[24] Guevara-Sada S, Laborde J, Sánchez-Ríos G (2004) Los Tuxtlas: El paisaje de la sierra. Instituto de Ecología AC and European Union.

[25] Cristóbal-Azkarate J, Veà J, Asensio N, Rodríguez-Luna E (2005) Biogeographical and floristic predictors of the presence and abundance of mantled howlers (*Alouatta palliata mexicana*) in rainforest fragments at Los Tuxtlas, Mexico. Am J Primatol 67: 209–222 10.1002/ajp.20178 16229005

[26] INEGI (2010) Censo de Población y Vivienda, 2010. Cuestionario básico.

[27] Velasco T (1997) Territorio e identidad chinanteca en Uxpanapa, Veracruz. In: Hoffmann O, Salmerón-Castro F, editors. Nueve Estudios Sobre el Espacio; Representación y Formas de Apropiación. Mexico City: CIESSAS. 133–153.

[28] Wasser SKS, Davenport B, Ramage ER, Hunt KE, Parker M, et al (2004) Scat detection dogs in wildlife research and management: application to grizzly and black bears in the Yellowhead Ecosystem, Alberta, Canada. Wildlife Biol 492: 475–492 10.1139/Z04-020

[29] Vynne C, Keim JL, Machado RB, Marinho-Filho J, Silveira L, et al (2011) Resource selection and its implications for wide-ranging mammals of the brazilian cerrado. PLoS One 6: e28939 10.1371/journal.pone.0028939 22205984 PMC3243687

[30] Wasser SK, Keim JL, Taper ML, Lele SR (2011) The influences of wolf predation, habitat loss, and human activity on caribou and moose in the Alberta oil sands. Front Ecol Environ 9: 546–551 10.1890/100071

[31] Ball MC, Pither R, Manseau M, Clark J, Petersen SD, et al (2007) Characterization of target nuclear DNA from faeces reduces technical issues associated with the assumptions of low-quality and quantity template. Conserv Genet 8: 577–586 10.1007/s10592-006-9193-y

[32] Haag T, Santos AS, De Angelo C, Srbek-Araujo AC, Sana DA, et al (2009) Development and testing of an optimized method for DNA-based identification of jaguar (*Panthera onca*) and puma (*Puma concolor*) faecal samples for use in ecological and genetic studies. Genetica 136: 505–512 10.1007/s10709-008-9347-6 19137401

[33] Michalski F, Valdez FP, Norris D, Zieminski C, Kashivakura CK, et al (2011) Successful carnivore identification with faecal DNA across a fragmented Amazonian landscape. Mol Ecol Resour 11: 862–871 10.1111/j.1755-0998.2011.03031.x 21676206

[34] Tamura K, Peterson D, Peterson N, Stecher G, Nei M, et al (2011) MEGA5: molecular evolutionary genetics analysis using maximum likelihood, evolutionary distance, and maximum parsimony methods. Mol Biol Evol 28: 2731–2739 10.1093/molbev/msr121 21546353 PMC3203626

[35] Vynne C, Skalski JR, Machado RB, Groom MJ, Jácomo AT, et al (2010) Effectiveness of scat-detection dogs in determining species presence in a tropical savanna landscape. Conserv Biol 25: 154–162 10.1111/j.1523-1739.2010.01581.x 21029162

[36] Över U, Gür D, Ünal S, Miller G (2001) Group A (2001) The changing nature of aminoglycoside resistance mechanisms and prevalence of newly recognized resistance mechanisms in Turkey. Clin Microbiol Infect 7: 470–478. 11678929 10.1046/j.1198-743x.2001.00284.x

[37] Díaz-Mejía JJ, Amábile-Cuevas CF, Rosas I, Souza V (2008) An analysis of the evolutionary relationships of integron integrases, with emphasis on the prevalence of class 1 integrons in *Escherichia coli* isolates from clinical and environmental origins. Microbiology 154: 94–102 10.1099/mic.0.2007/008649-0 18174129

[38] Roe M, Vega E, Pillai S (2003) Antimicrobial resistance markers of class 1 and class 2 integron-bearing *Escherichia coli* from irrigation water and sediments. Emerg Infect Dis 9: 822–826. 12890322 10.3201/eid0907.020529 PMC3023436

[39] Rempel R, Elkie P (1999) Patch Analyst 2.2. Center for Northern Forest Ecosystem Research. Available: http://flash.lakeheadu.ca/~rrempel/ecology/.

[40] Garrec H, Drieux-Rouzet L, Golmard J-L, Jarlier V, Robert J (2011) Comparison of nine phenotypic methods for detection of extended-spectrum beta-lactamase production by Enterobacteriaceae. J Clin Microbiol 49: 1048–1057 10.1128/JCM.02130-10 21248086 PMC3067698

[41] Martinez JL (2009) Environmental pollution by antibiotics and by antibiotic resistance determinants. Environ Pollut 157: 2893–2902. 19560847 10.1016/j.envpol.2009.05.051

[42] Mandujano S, Escobedo-Morales LA, Palacios-Silva R (2004) Movements of *Alouatta Palliata* among forest fragments in Los Tuxtlas, Mexico. Neotrop Primates 12: 126–131.

[43] Chaves OM, Stoner KE (2010) River crossings by *Ateles geoffroyi* and *Alouatta pigra* in southern Mexico: A preliminary report. Rev Chil Hist Nat 83: 435–442.

[44] Amábile-Cuevas CF, Arredondo-García JL, Cruz A, Rosas I (2010) Fluoroquinolone resistance in clinical and environmental isolates of *Escherichia coli* in Mexico City. J Appl Microbiol 108: 158–162 10.1111/j.1365-2672.2009.04401.x 19548885

[45] Bonnedahl J, Hernandez J, Stedt J, Waldenström J, Olsen B, et al (2014) Extended-spectrum β-lactamases in *Escherichia coli* and *Klebsiella pneumoniae* in gulls, Alaska, USA. Emerg Infect Dis 20: 897–899. 24750592 10.3201/eid2005.130325 PMC4012786

[46] Stedt J, Bonnedahl J, Hernandez J, McMahon BJ, Hasan B, et al. (2014) Antibiotic resistance patterns in *Escherichia coli* from gulls in nine European countries. Infect Ecol Epidemiol 4. doi:10.3402/iee.v.4.21565. 10.3402/iee.v4.21565 PMC3889177 24427451

[47] Bildstein KL (2004) Raptor migration in the Neotropics: patterns, processes, and consequences. Ornitol Neotrop 15: 83–99.

[48] DiFiore A, Campbell CJ (2007) The atelines: Variation in ecology, behaviour, and social organization. In: Campbell CJ, Fuentes A, MacKinnon KC, Panger M, Bearder SK, editors. Primates in perspective. Oxford: Oxford University Press.

[49] Amábile-Cuevas CF (2013) Antibiotic resistance: from Darwin to Lederberg to Keynes. Microb Drug Resist 19: 73–87 10.1089/mdr.2012.0115 23046150

